# Diet Type Impacts Production Performance of Fattening Lambs by Manipulating the Ruminal Microbiota and Metabolome

**DOI:** 10.3389/fmicb.2022.824001

**Published:** 2022-04-25

**Authors:** Siqi Li, Meiyu Du, Chongyu Zhang, Yun Wang, Yunkyoung Lee, Guiguo Zhang

**Affiliations:** ^1^College of Animal Sciences and Technology, Shandong Agricultural University, Taian, China; ^2^Interdisciplinary Graduate Program in Advanced Convergence Technology and Science, Department of Food Science and Nutrition, Jeju National University, Jeju, South Korea

**Keywords:** lambs, dietary forms, microbiome, metabolome, performance

## Abstract

The pelleted total mixed ration (PTMR) has a positive effect on the productivity of fattening lambs. However, whether the beneficial effects are underpinned by altering the rumen microbiota and metabolome that remain unclear. This study aimed to investigate correlations among growth performance, ruminal microbiota, and ruminal metabolome of lambs fed PTMR diet. A total of 100 crossbred (Dorper sheep × Fine-wool sheep) ram lambs at 55 days of age with similar body weight (BW) (13.2 ± 0.5 kg) were randomly allocated to 10 pens that were fed either PTMR (PTMR group) or unpelleted total mixed ration (UPTMR group) with the same dietary ingredients and nutritional contents. The average daily gain (ADG) and average daily feed intake (ADFI) were determined during the 62-day experimental period and ruminal pH, volatile fatty acid (VFA) concentrations, microbiota, and metabolome in the rumen of the lambs were examined at the end of the experiment. Compared to those of the UPTMR group, the PTMR group had greater ADFI (*P* = 0.002), ADG (*P* = 0.003), and feed efficiency (G/F) (*P* < 0.05). Similarly, feeding PTMR increased the concentration of total VFA (TVFA) and the molar proportion of propionate, but decreased the proportion of butyrate and acetate to propionate ratio in the rumen of lambs compared to that in lambs from the UPTMR group (*P* < 0.05). In addition, the PTMR group demonstrated lowered alpha-diversity of the ruminal microbiota and enhanced the relative abundance of *Fibrobacter* (*P* < 0.05), *Veillonellaceae* (*P* < 0.05), and the abundance of *Rikenellaceae* (*P* = 0.064) in the rumen compared with those in the UPTMR group. Feeding lambs with PTMR significantly upregulated the metabolic pathways involving tryptophan, histidine, cysteine and methionine, β-alanine, tyrosine metabolisms, and steroid biosynthesis. Moreover, the abundance of the microbiota strongly correlated with the altered performance, ruminal VFA, metabolites, and metabolic pathways of lambs. Taken together, feeding PTMR shaped the ruminal microbiota of lambs with decreased diversity, while improving relative abundance of some specific microbes and upregulating certain growth-related metabolic pathways, which contributed to the augmented growth performance and G/F of fattening lambs. Thus, feeding PTMR to fattening lambs for superior production performance and G/F is recommended.

## Introduction

The high growth performance of feedlot lambs relies on a stable supply of nutrition-balanced feed (Zhang et al., 2019). However, the deficiency of high-quality forage has severely restricted the development of intensive rearing system for lambs in farming areas (Newell et al., 2020; Larsen et al., 2021). Due to the shortage of land and seasonal oscillations, it has become difficult to provide abundant and stable forage for livestock feeding (Yang et al., 2018; Shen et al., 2020); low-quality crop stalks constitute the main source of roughage in the fattening lamb diet, which significantly impede lambs from achieving their productivity potential (Blanco et al., 2014). Therefore, exploring workable strategies, improving the roughage efficiency, and establishing the stable feed supply system for promoting the development of the fattening lamb industry are important. Diet characteristics, such as ingredients, shape, smell, particle size, and processing methods, affect G/F and animal feed intake (Bi et al., 2019; Ran et al., 2021; Zhang et al., 2021). The pelleted total mixed ration (PTMR) has been shown to enhance the G/F and growth performance of lambs (Zhang et al., 2019; Li et al., 2021). The pelleting process includes a steam heat-treated conditioning technique (Tran et al., 2007; Abdollahi et al., 2010) and applying mechanical pressure, which can partly breakdown complex fiber structures and promote starch gelatinization resulting in an increased feed palatability and nutritional digestibility (Bo Trabi et al., 2020). Additionally, compared to the traditional feeding pattern of separated concentrate and roughage or unpelleted total mixed ration (UPTMR), feeding the PTMR diet to lambs reshapes the differentiated gastrointestinal microbial community, which is positively correlated to productivities of lambs (Zhang et al., 2019; Li et al., 2021). Similarly, other studies have revealed that components and abundance of ruminal microbiota are tightly linked to the productivity of ruminants (Wallace et al., 2019) and analyzing the ruminal microbiota genes and species can accurately predict the animals’ G/F (Shabat et al., 2016; Du et al., 2021). Diet drives gastrointestinal microbiota composition and metabolism, linking microbes to diet and host physiological status and phenotypes (Koh et al., 2016; Liu et al., 2021). The enhanced nutrient digestibility of the diet is closely related to the increased abundance of specific ruminal microbes and regulating metabolic pathways (Bo Trabi et al., 2020; Gebeyew et al., 2021), rather than increased overall diversity (Shabat et al., 2016). One of the mechanisms by which the microbiota affects the host’s phenotypes is by producing harmful metabolites associated with the development of disease or beneficial metabolites that promote growth performance (Koh et al., 2016; Liu et al., 2021).

Thus, ruminal microbiota may be a potential target that can be manipulated by an appropriate diet strategy to produce beneficial metabolites, thereby facilitating improved performance (Liu et al., 2021). Achieving a better understanding of the mechanisms by which ruminal bacteria interact with diet should facilitate developing the appropriate dietary strategies not only to reshape a health-promoting microbiota, but also to improve growth performance (Ran et al., 2021). The PTMR has been reported to increase the productivity and the G/F of lambs (Zhang et al., 2019; Li et al., 2021). Similarly, we have previously observed that feeding lambs with PTMR reconditioned the structure and composition of ruminal microbiota in fattening lambs, which contributed to the improved ruminal fermentation and growth performance of fattening lambs (Zhang et al., 2019).

However, few studies have evaluated the effects of feeding PTMR to lambs on ruminal metabolome and metabolic pathways of lambs. Meanwhile, it is still unclear whether there is a close relationship among the alterations of performance, microbiota, metabolome, and metabolic pathway of lambs fed PTMR diet.

It is hypothesized that feeding PTMR to lambs impacting the growth performance through modulating the ruminal microbiota, metabolome, and growth-related metabolic pathways. The objectives of this study were to evaluate the effects of feeding PTMR or UPTMR to lambs on the growth performance, ruminal microbiota, and metabolites and whether there is the close association among them.

## Materials and Methods

All the procedures involving Animal Care and Use were in strict accordance with the Animal Care and Use Protocol approved by the Shandong Agricultural University Animal Nutrition Research Institute (Protocol No. 20190316).

### Preparation of Experimental Pelleted Total Mixed Ration

The experimental diets were formulated to meet the nutrient requirements recommended by the Feeding Standard of Sheep of the People’s Republic of China (NY/T 816-2004). Dietary composition and nutrient content are given in Supplementary Table 1. Roughages in the formula and all the concentrates were ground to pass through 3- and 1.5-mm screen, respectively. The PTMR diet was produced according to the procedure described by Zhang et al. (2019) with some minor modification for high-quality pellets. Briefly, after dietary components were mixed thoroughly, the PTMR diet was pelleted at the 70–85°C with a compression ratio of 10:1 to form a cylindrical shape (pellet diameter 0.8 mm; length 10–15 mm) using a pelleting machine (FSP56 × 40, MuYang Corporation Ltd., China). All the PTMR diets were prepared in one batch and stored in covered containers, whereas UPTMR was mixed every 2 days to maintain freshness.

### Experimental Design, Animals, and Feeding Management

In total 100 healthy crossbred ram lambs (Dorper sheep × Fine-wool sheep) at 55-day old with similar body weight (BW) (13.2 ± 0.5 kg) were randomly allocated into 10 pens with 10 lambs in each pen (3 m × 10 m). Pens were randomly assigned to two dietary treatments with five replicates per treatment. The two dietary treatments were PTMR and UPTMR. The experiment consisted of a 10-day acclimatization period and a 62-day data collection period (Supplementary Figure 1). Lambs were fed twice daily (at 06:00 and 18:00 h) to allow *ad libitum* intake and had free access to water through fresh tap water. During the experimental period, all the lambs were weighed at 65, 78, 97, 113, and 126 days of age before morning feeding to determine average daily gain (ADG). The ADG was calculated for four intervals: 65–78, 78–97, 97–113, and 113–126 days of age and the change in weight for each interval was divided by the number of days for each interval. The daily feed offered, orts, and spillages were collected and weighed to calculate the average daily feed intake (ADFI). G/F was expressed as the ratio of ADG to ADFI (ADG/ADFI, G/F).

### Sample Collection and Determination

At the end of the experiment, 10 healthy fattening lambs (1 from each pen, 5 for each treatment) were randomly selected and slaughtered using electrical stunning according to the procedures recommended by the Animal Ethics Committee at Shandong Agricultural University. After removal of the skin, head, and feet, as described by Zhang et al. (2019), the abdominal cavity was immediately opened. A small hole was cut in the rumen wall using a sterile sharp surgical knife and a pH probe (PHBJ-260, INESA, Shanghai, China) was placed into the luminal contents to measure ruminal pH. The rumen was subsequently dissected from the digestive tract using a sterile scalpel. A 250 ml mixed rumen content (containing liquid and solid) was collected and immediately stored in liquid nitrogen or −80°C refrigerator for further determination of ruminal volatile fatty acid (VFA), microbiota, and metabolome.

### Determination of Volatile Fatty Acid in the Rumen Digesta

The collected ruminal digesta sample (the whole digesta including solid and liquid mixture) was strained through four layers of cheesecloth and centrifuged at 15,000 × *g* for 15 min at 4°C. A 10 ml supernatant was drawn to analyze VFA concentrations using high-performance gas chromatography (GC97, FULI, Zhejiang) following the procedure described by Zhong et al. (2018) and Wang et al. (2019). Briefly, the sample supernatant was injected into an HP UF-FFAP-M3025 capillary column. A flame ionization detector was used to quantify VFA content. The oven temperature was set at 140°C. The injector and detector temperatures were set at 250 and 260°C, respectively, and the nitrogen flow was 50 ml/min.

### Ruminal Microbiota Determination by 16S rDNA Amplicon Sequencing

Samples of collected rumen whole digesta taken after slaughter were stored in 5 ml cryogenic vials and immediately flash frozen in liquid nitrogen until analysis. DNA from rumen whole digesta samples was extracted using the QIAamp DNA Stool Mini Kit (Qiagen Incorporation, Hilden, Germany) according to the manufacturer’s instructions. The V3–V4 hypervariable region of the 16S rRNA gene was amplified with universal primers 515F (GTGCCAGCMGCCGCGGTAA) and 806R (GGACTACHVGGGTWTCTAAT) as described by Passetti et al. (2020). Amplicon libraries were sequenced on the Ion S5™ XL sequencing platform (Novogene, Beijing, China) for single-end reads of 400 and 600 bp (SE400 and SE600). The raw single-end reads were assembled into longer sequences and quantitatively filtered using PANDAseq (version 2.9) to remove the low-quality reads with a length of <220 nucleotides (nt) or >500 nt, an average quality score of <20, and sequences containing >3 nitrogenous bases. The high-quality sequences were clustered into operational taxonomic units (OTUs) with 97% similarity using UPARSE (version 7.0) in QIIME (version 1.8) (Caporaso et al., 2011) and the chimeric sequences were removed using UCHIME. Taxonomy was assigned to OTUs using the RDP Classifier 1 (Bacci et al., 2015) against the SILVA 16S rRNA gene database (Release 1282), with a confidence threshold of 70%. All the raw data involved in this study were deposited in the National Center for Biotechnology Information (NCBI) Sequence Read Archive (SRA) under accession number SUB7211899. The Shannon diversity index and the number of OTUs per sample were calculated using the Mothur program (version 1.30.13). Bar plots and heat maps were generated using the heatmap illustrator program (version 1.0.3.7). For beta-diversity analysis, principal component analysis (PCoA) was performed based on weighted UniFrac distances using SIMCA (version 14.1).

### Ruminal Metabolome Determination by Liquid Chromatography–Tandem Mass Spectrometry

The metabolome of ruminal digesta was determined through Novogene Corporation Ltd., Beijing, China, following the processing program described by Cao et al. (2020, 2021).

### Metabolite Extraction

The frozen rumen samples were individually grounded with liquid nitrogen and the homogenate was resuspended with prechilled 80% methanol. The samples were incubated on ice for 5 min and then were centrifuged at 15,000 × *g*, 4°C for 20 min. Supernatant was diluted to final concentration of 53% methanol by liquid chromatography–tandem mass spectrometry (LC-MS) grade water. The samples were subsequently transferred to a fresh tube and then were centrifuged at 15,000 × *g*, 4°C for 20 min and the resultant supernatant was analyzed using LC–MS/MS as described below.

### Ultra-High-Performance Liquid Chromatography–Tandem Mass Spectrometry Analysis

Liquid chromatography–tandem mass spectrometry analyses were performed using the Vanquish UHPLC system (Thermo Fisher Scientific, Shanghai, China) coupled with the Orbitrap Q Exactive series mass spectrometer (Thermo Fisher Scientific, Shanghai, China). A Hypersil Gold HPLC column (Thermo Fisher Scientific; 100 mm × 2.1 mm, 1.9 μm) with a 16-min linear gradient and 0.2 ml/min flow rate was employed. Eluent A (0.1% formic acid in water) and eluent B (methanol) were used for positive polarity mode. Eluent A (5 mM ammonium acetate, pH 9.0) and eluent B (methanol) were used for negative polarity mode. The following solvent gradient was used: 2% eluent B, 1.5 min; 2–100% eluent B, 12.0 min; 100% eluent B, 14.0 min; 100–2% eluent B, 14.1 min; and 2% eluent B, 17 min. The mass spectrometer was operated in positive/negative polarity mode with the following settings: spray voltage = 3.2 kV, capillary temperature = 320°C, sheath gas flow rate = 35 arbitrary unit (arb), and auxiliary gas flow rate = 10 arb.

### Data Processing and Metabolite Identification

The raw data files generated by ultra-high-performance liquid chromatography–tandem mass spectrometry (UHPLC-MS/MS) were processed using the Compound Discoverer 3.1 (CD3.1, Thermo Fisher Scientific, Waltham, MA, United States) to perform peak alignment, peak picking, and quantitation for each metabolite. The main parameters were set as follows: retention time tolerance, 0.2 min; actual mass tolerance, 5 ppm; signal intensity tolerance, 30%; signal/noise ratio, 3; and minimum intensity = 104. Peak intensities were normalized to the total spectral intensity. The normalized data were used to predict the molecular formula based on additive ions, molecular ion peaks, and fragment ions. Then, peaks were matched with the ChemSpider, mzCloud, and mzVault databases to obtain the accurate qualitative and relative quantitative results and all the compounds were annotated in level 3 following the Metabolomics Standards Initiative definition (Sumner et al., 2007). In this study, all the compounds were fully matched in the ChemSpider database.

### Calculations and Statistical Analysis

In this study, a pen was the experimental unit for growth performance measurements (*n* = 5) and individual lamb was used as the experimental unit for ruminal fermentation characteristics (*n* = 5) and microbiota and metabolome analysis (*n* = 5). Data for ADFI, ADG, and G/F were analyzed using the MIXED procedure for repeated measures in the IBM SPSS Statistics version 23 with treatment, period, and the interaction between treatment and period as fixed effects. Period was the repeated measurement and pen nest within treatment was the subject of repeated measurements. An autoregressive covariance was included in the model to adjust the time effect. BW and ruminal fermentation characteristics were examined using the one-way ANOVA in the GLM procedure. The significance of differences between the two treatments was tested using LSMEANS with the PDIFF option. Linear discriminant analysis (LDA) effect size analysis of ruminal microbiota changes was conducted using the online procedure of Galax.^1^ Differences were considered as statistically significant when *P* < 0.05. To compare the distribution of the ruminal metabolites of the two groups, the orthogonal projections to latent structures discriminant analysis (OPLS-DA) was conducted with SIMCA14.1. The enrichment of metabolic pathways and the analysis of discrepant metabolic pathways were conducted using MetaboAnalyst version 4.0 online procedure and the *Bos taurus* Kyoto Encyclopedia of Genes and Genomes (KEGG) pathway database.^2^ The Spearman’s rank correlation analysis was performed to evaluate the potential link between alterations in performance, ruminal microbiota, metabolites, and modified metabolic pathways of fattening lambs.

## Results

### Effects of Dietary Types on Growth Performance of Fattening Lambs

The effects of dietary types on BW, ADG, ADFI, and G/F of lambs are shown in Table 1. A similar initial BW (*P* > 0.05) of lambs was observed between the PTMR and UPTMR groups. After 32 days (age = 97 days), lambs from the PTMR group had greater BW (*P* < 0.05) compared to that of the UPTMR group. In addition, at four time points of 78, 97, 113, and 126 day-old, the PTMR-fed lambs had an increased ADFI (*P* < 0.05) than those fed the UPTMR diet, suggesting an increased feed intake. Moreover, compared to those of the UPTMR group, the PTMR-fed lambs had an increased ADG (*P* < 0.05) and G/F during the entire experimental period (age of 65–126 days).

**TABLE 1:** Effects of dietary types on productivity of fattening lambs.

Items	Treatments^1^		SEM	*P*-value
	
	PTMR^1^	UPTMR		
**Body weight (kg)**				
Day 65	17.2	17.1	0.286	0.411
Day 78	20.9	20.2	0.307	0.236
Day 97	26.6	25.1	0.269	0.024
Day 113	31.4	29.6	0.238	0.005
Day 126	35.4	33.4	0.241	0.003
**Day 65–78**				
ADG^2^ (g/day)	291	238	3.2	0.008
ADFI (kg/day)	0.97	0.92	0.004	0.001
G:F	0.29	0.25	0.053	0.229
**Day 78–97**				
ADG (g/day)	295	258	9.8	0.092
ADFI (kg/day)	1.11	1.03	0.005	0.001
G:F	0.27	0.25	0.036	0.305
**Day 97–113**				
ADG (g/day)	301	282	6.6	0.183
ADFI (kg/day)	1.24	1.19	0.008	0.018
G:F	0.24	0.24	0.084	0.435
**Day 113–126**				
ADG (g/day)	311	295	3.2	0.038
ADFI (kg/day)	1.42	1.39	0.015	0.023
G:F	0.22	0.21	0.047	0.120
**Day 65–126**				
ADG (g/day)	292	268	2.4	0.001
ADFI (kg/day)	1.18	1.13	0.001	0.001
G:F	0.25	0.24	0.014	0.035

*^1^PTMR, pelleted total mixed ration; UPTMR, unpelleted total mixed ration. ^2^ADG, average daily gain; ADFI, average daily feed intake; feed efficiency was calculated by dividing ADG by ADFI (G:F).*

### Effects of Dietary Types on Rumen Fermentable Parameters of Fattening Lambs

The effects of the two feeding regimes on ruminal fermentation characteristics are given in Table 2. Compared to the UPTMR diet, PTMR increased the concentration of TVFA (*P* < 0.01), the ratio of propionate (*P* < 0.01), and decreased the percentage of butyrate (*P* = 0.003) and the ratio of acetate to propionate (*P* = 0.004). However, no significant differences in ruminal pH values (*P* = 0.576) and acetate proportion (*P* = 0.583) were observed between the PTMR and UPTMR groups.

**TABLE 2:** Effects of dietary types on rumen fermentable parameters of fattening lambs.

Item	Treatments^1^		SEM	*P*-values
	
	PTMP	UPTMR		
TVFA^2^, mM	107.60	92.51	8.200	<0.01
Ruminal pH	6.05	6.16	0.095	0.576
Acetate, mol/100 mol	60.55	60.74	0.496	0.583
Propionate, mol/100 mol	27.26	26.25	0.574	<0.01
Butyrate, mol/100 mol	12.18	13.00	0.531	<0.01
Acetate: propionate	2.21	2.31	0.060	<0.01

*^1^PTMR, pelleted total mixed ration; UPTMR, unpelleted total mixed ration. ^2^TVFA, total volatile fatty acids.*

### Profile and Characteristics of the Ruminal Microbial Community of Lambs Fed Pelleted Total Mixed Ration or Unpelleted Total Mixed Ration

**FIGURE 1:**
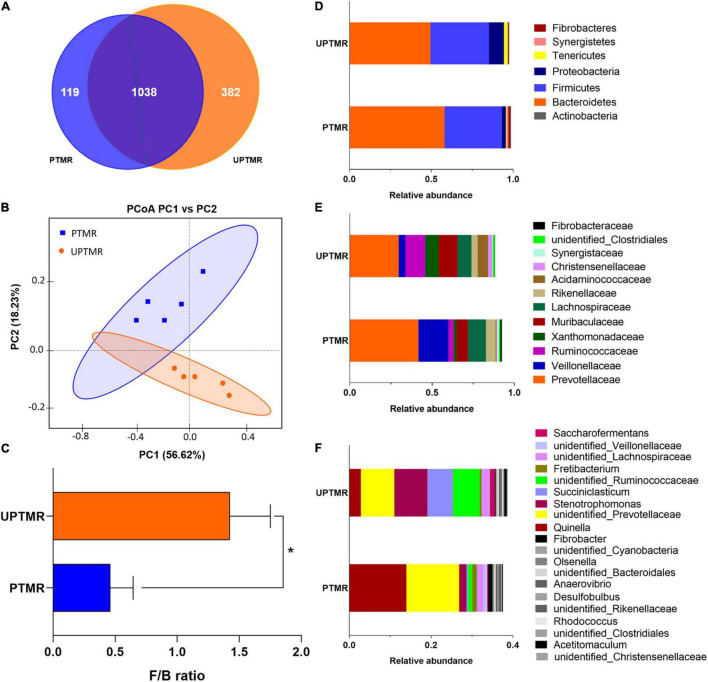
The operational taxonomic unit (OTU) numbers, beta-diversity [principal coordinate analysis (PCoA) plot], and the relative abundances at the phylum, family, and genus levels of the ruminal microbiota of lambs fed pelleted total mixed ration (PTMR) or unpelleted PTMR (UPTMR) diet. **(A)** Venn diagram of OTUs in the ruminal microbiota. **(B)** Principal coordinate analysis (PCoA). **(C)** The ratio of *Firmicutes* and *Bacteroidetes*. The relative abundances at the **(D)** phylum, **(E)** family, and **(F)** genus levels of ruminal microbiota of lambs fed PTMR or UPTMR.

A total of 73,006 and 83,024 raw reads were generated in the PTMR and UPTMR groups, respectively. After removing low-quality sequences, 66,012 and 77,204 total tags in the rumen contents for the PTMR and UPTMR groups were obtained, respectively. Based on 97% sequence similarity, a total of 1,157 OTUs were identified in the PTMR group, which were then assigned to 22 phyla, 30 classes, 53 orders, 83 families, and 128 genera. In the UPTMR group, 1,420 OTUs were obtained and were clustered into 25 phyla, 36 classes, 65 orders, 96 families, and 152 genera. There were 1,038 OTUs that were common across the two experimental groups (Figure 1A).

The sequencing depth was reflected by the richness of total microbial species (good coverage >99%) and the majority of OTUs were exerted at a low richness, as demonstrated by the rarefaction, rank abundance, Shannon index, and PD_whole_tree curves. The PD_whole_tree, Shannon indexes, and Simpson indices were greater in the UPTMR group (*P* < 0.01) than those in the PTMR group. Similarly, increased Chao1, observed species, and ACE values were observed in the UPTMR group compared to those in the PTMR group. This displayed a greater alpha-diversity and richness of the ruminal microbial community in the UPTMR group than that in the PTMR group (Table 3). In addition, PCoA revealed that the microbial community structures of the rumen were almost completely separated because of the TMR-type discrepancy (Figure 1B).

**TABLE 3:** Alpha-diversity indices of the ruminal microbiota of lambs fed different types of total mixed ration (TMR).

Item	Coverage^1^,%	Richness estimators			Alpha-diversity indexes		
			
		OS	Chao1	ACE	PD_whole_tree	Shannon	Simpson
PTMR	>99	712	866.71	894.58	57.93	5.57	0.9
UPTMR	>99	932	1062.20	1075.04	70.74	7.12	0.97
SEM		21.3	19.28	16.42	1.79	0.21	0.02
*P*-value		0.001	0.001	0.001	0.007	0.006	0.106

*^1^Coverage percentage; richness estimators represented by Chao1, observed species (OS), and ACE; alpha-diversity indices included the PD_whole_tree, Shannon, and Simpson.*

The relative abundance of ruminal microbiota that occurred at more than 0.5% was determined at the phylum, family, and genus levels (Figures 1D–F and Supplementary Table 2). The ruminal microbiota in both the PTMR and UPTMR groups were both dominated by the phyla *Bacteroidetes* and *Firmicutes*. The proportions of *Bacteroidetes* richness were 0.58 and 0.49 and those of the *Firmicutes* accounted for 0.35 and 0.36 of the total abundance in the PTMR and UPTMR groups, respectively. In addition, the abundance of *Proteobacteria* was 9.19% in the UPTMR group, which was higher (*P* < 0.05) than that in the PTMR group (2.54%). The dominant families within the phylum *Bacteroidetes* consisted of *Prevotellaceae*, *Rikenellaceae*, and *Bacteroidaceae*. The main families within the phylum *Firmicutes* were *Ruminococcaceae*, *Erysipelotrichaceae*, *Lachnospiraceae*, *Veillonellaceae*, and *Acidaminococcaceae*. Other phyla (*Cyanobacteria*, *Tenericutes*, *Actinobacteria*, *Synergistetes*, and *Fibrobacteres*) were present in relatively low abundance. The main microbial communities in both the PTMR and UPTMR groups were *Quinella*, *Prevotellaceae*, *Ruminococcaceae*, *Succiniclasticum*, *Saccharofermentans*, *Veillonellaceae*, *Fibrobacter*, and *Rikenellaceae*.

### Discrepant Bacterial Communities in Ruminal Microbiota of Lambs Fed Pelleted Total Mixed Ration or Unpelleted Total Mixed Ration Diet

**FIGURE 2:**
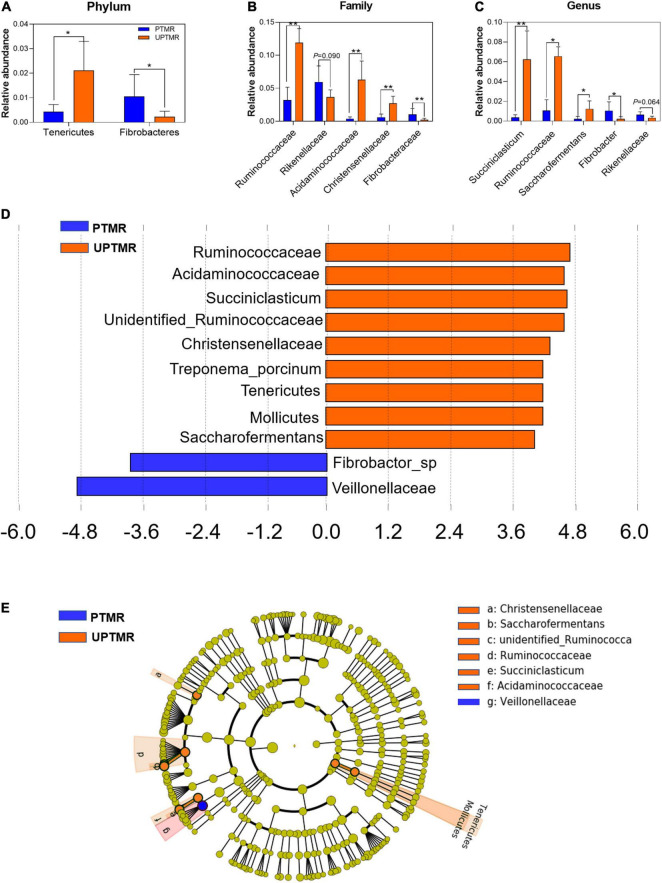
The relative abundances of ruminal microbiota that were significantly different between the LE and HE groups at the **(A)** phylum, **(B)** family, and **(C)** genus levels. Only microbes that had a relative abundance of more than 0.1% were compared. Linear discriminant analysis (LDA) value distributed histogram and cladogram of different microorganisms (LDA score >3.5). **(D)** Linear discriminant analysis (LDA) value distributed histogram. **(E)** Cladogram constructed to visualize the microbial community relative abundance data of ruminal samples between the PTMR and UPTMR groups. Different color nodes indicate different groups and the species classifications at the phylum, class, order, family, and genus levels are shown from the inside to the outside. Difference was declared to be statistically significant when *P < 0.05, **P < 0.01.

Alterations in microbial communities by the diet between the PTMR and UPTMR groups at the phylum, family, and genus levels are shown in Figures 1, 2. In the rumen, abundance analysis of the main microbiota with high abundance indicated that feeding PTMR to lambs decreased (*P* < 0.05) the F/B ratio, while increased (*P* < 0.05) the relative abundance of phylum *Fibrobacteres* compared to those lambs fed UPTMR diet (Figure 1C). In addition, the relative abundance of *Tenericutes* increased (*P* < 0.05) in the UPTMR group compared to that in the PTMR group (Figure 2A). The microbial communities were further compared at the family level. The relative abundance of *Ruminococcaceae*, *Acidaminococcaceae*, and *Christensenellaceae* in the PTMR group (3.24, 0.40, and 0.58%) decreased (*P* < 0.001, *P* = 0.002, and *P* = 0.003, respectively) compared to that in the UPTMR group (11.95, 6.29, and 2.50%, respectively). In contrast, feeding lambs with PTMR increased (*P* < 0.05) the relative abundance of *Rikenellaceae* and *Fibrobacteraceae* compared to the UPTMR group. Microbial communities were further compared at the genus level. The relative abundance of *Ruminococcaceae*, *Succiniclasticum*, and *Saccharofermentans* significantly increased (*P* < 0.001) in the UPTMR group compared to the PTMR group (Figure 2A), whereas the PTMR group had a greater (*P* < 0.05) relative abundance of *Fibrobacteria* and *Rikenellaceae* compared with the UPTMR group.

Linear discriminant analysis effect size (LefSe) analysis found different effects of TMR feeding on ruminal microbiota in lambs (Figures 2B,C). The results showed that feeding PTMR increased the relative abundance of *Fibrobacter* and *Veillonellaceae* (LDA score >3.5) and decreased the richness of *Ruminococcaceae*, *Succiniclasticum*, *Saccharofermentans*, *Acetitomaculum*, and *Christensenellaceae* (LDA score >3.5) in the rumen compared to the UPTMR-fed group (Figure 2B). The cladograms displayed the phylogenetic distribution of discrepant bacteria in the PTMR and UPTMR groups (Figure 2C).

### Profile of Ruminal Metabolites and Enrichment of Metabolic Pathways

**FIGURE 3:**
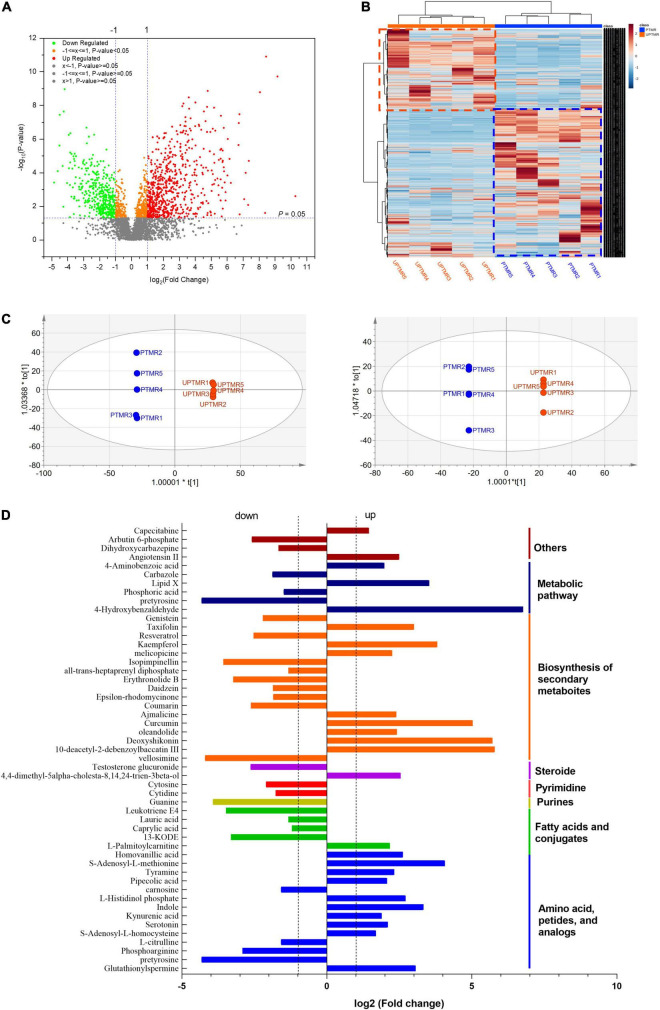
**(A)** Volcano plot shown the profile of metabolites and discrepant metabolites between the PTMR and UPTMR groups. **(B)** Heatmap indicated effects of the dietary form (PTMR vs. UPTMR) on the ruminal metabolites of lambs. **(C)** The orthogonal projections to latent structures discriminant analysis (OPLS-DA) to the ruminal metabolites of the PTMR and UPTMR groups. **(D)** The classifications of discrepant metabolites (*P* < 0.05) with upregulated or downregulated in the PTMR group compared with those of the UPTMR group.

The analysis of ruminal content metabolome determined by LC–MS displayed that 3,548 metabolites (positive and negative ions, Supplementary Table 3) were detected in the two groups, of which 758 differentiated metabolites (log2FC >1.2, *P* < 0.05) were identified (Figure 3A). The correlation analysis (Figure 3B, heatmap) indicated that the metabolites from the biological duplication samples of the PTMR or UPTMR group clustered together well and the varieties of metabolites were correlated tightly with the dietary treatment. To further compare the distribution of the ruminal metabolites of the two groups, OPLS-DA was conducted (Figure 3C). The results further displayed a completely separated clustering between PTMR and UPTMR, suggesting that rumen metabolites were typically modified by the two forms of the TMR diet. The classification analysis of significantly modulated metabolites revealed that those regulated metabolites by dietary form mainly included amino acids, fatty acids, purines, pyrimidines, steroids, and secondary metabolites. Fifty-two primary differential metabolites and their categories are given in Figure 3D.

### Correlation Between Growth Performance, Ruminal Bacteria, and Metabolites

**FIGURE 4:**
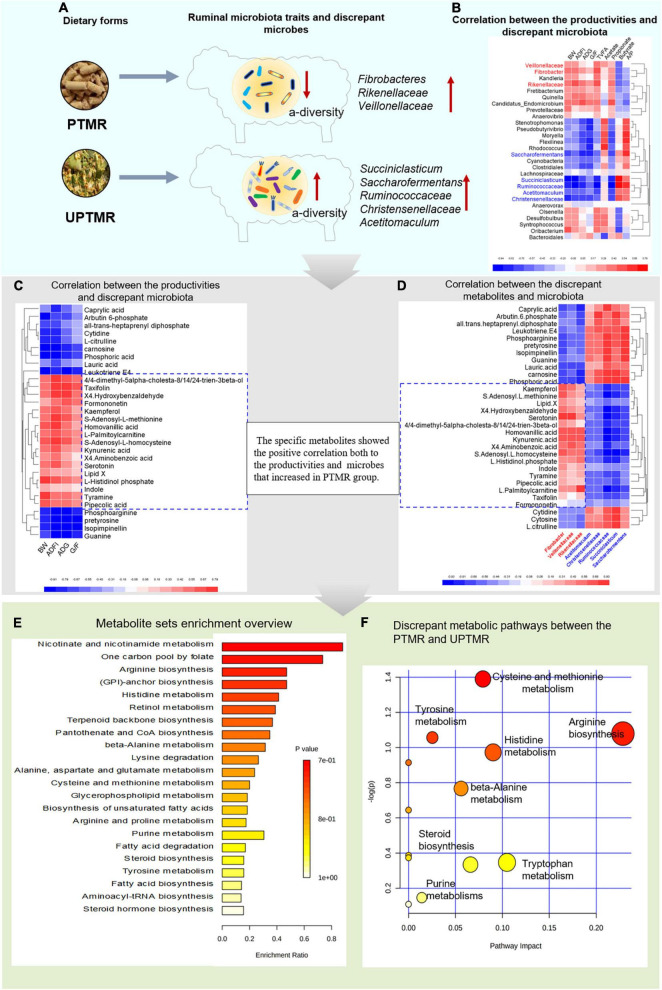
The cause-and-effect process of dietary form affecting the performance of lambs fed PTMR or UPTMR diet. **(A)** The dietary form specially impacted the ruminal microbes. **(B)** Correlation between the production performance [body weight (BW), average daily gain (ADG), average daily feed intake (ADFI), and G/F)], volatile fatty acid (VFA) yield, and differentiated ruminal microbiota. **(C)** Correlation between the differentiated ruminal microbiota and metabolites. **(D)** Correlation between the growth performance and differential metabolites. **(E)** Enriched metabolic pathways based on the differentiated metabolites between the PTMR and UPTMR groups, following the library of pathway-associated metabolites sets (SMPDB). *P*-value showed the match status and *P* < 0.05 means a well matching. **(F)** Pathway analysis of differential metabolites. The manipulated metabolic pathways are based on the analysis of differentiated ruminal metabolites of lambs fed PTMR or UPTMR diets following the *Bos taurus* Kyoto Encyclopedia of Genes and Genomes (KEGG) pathway database. The metabolome view shows all the matched pathways according to the *P*-values from the pathway enrichment analysis and impact values from the topology analysis. The node colors varied from yellow to red, indicating that the metabolites have the data with different levels of significance. 1, arginine biosynthesis; 2, cysteine and methionine metabolism; 3, histidine metabolism; 4, tyrosine metabolism; 5, tryptophan metabolism; 6, malate–aspartate shuttle; 7, steroid biosynthesis; and 8, purine metabolism.

The analysis of the Spearman’s rank correlation and metabolic pathways revealed that 43 metabolic pathways were enriched, mainly referring to the growth-related amino acid metabolism, fatty acid metabolism, and steroid biosynthesis, by matching 758 different metabolites with the database of pathway-associated metabolite sets (SMPDB). These modified metabolic pathways further revealed the underlying mechanism by which dietary form affected the performance, G/F, and ruminal fermentation. The visualized findings are presented in a heat map (Figure 4). The diet regimes (PTMR vs. UPTMR) specifically modified the abundance of certain microbes (Figure 4A). In particular, the genera *Fibrobacteres*, *Rikenellaceae*, and *Veillonellaceae* with increased relative abundance in the PTMR group were positively correlated with BW, ADG, ADFI, G/F, TVFA yield, and propionate ratio (*P* < 0.05) (Figure 4B, red font). In contrast, the genera *Ruminococcaceae*, *Christensenellaceae*, *Succiniclasticum*, *Saccharofermentans*, and *Acetitomaculum*, with an increased abundance in the UPTMR group, were negatively correlated with alterations in BW, ADG, ADFI, G/F, TVFA yield, and propionate ratio (*P* < 0.05; Figure 4B).

In addition, the genera *Fibrobacter*, *Rikenellaceae*, and *Veillonellaceae*, which had an increased abundance in the PTMR group, exhibited a positive correlation with certain specific ruminal metabolites, including *S*-adenosyl-L-methionine, *S*-adenosyl-L-homocysteine, serotonin, kynurenic acid, indole, 4,4-dimethyl-5alpha-cholesta-8,14,24-trien-3beta-ol, L-histidinol phosphate, tyramine, and homovanillic acid, while negatively correlated with carnosine, guanine, phosphoarginine, and L-citrulline (*P* < 0.05; Figure 4C).

Moreover, the modified metabolites were positively correlated with BW, ADG, ADFI, and G/F of lambs (*P* < 0.01; Figure 4D) and were further enriched into 13 growth-related metabolic pathways according to the *P*-values from the pathway enrichment analysis and impact values from the pathway topology analysis following the *Bos taurus* KEGG pathway database (Figures 4E,F). Of these, five differential metabolic pathways were upregulated in the PTMR groups, including the metabolism of tryptophan, histidine, tyrosine, cysteine and methionine, and steroid biosynthesis, whereas three metabolic pathways were upregulated in the UPTMR groups in terms of arginine biosynthesis, beta-alanine metabolism, and purine metabolism (Figures 4E,F).

## Discussion

The intensive feedlot rearing pattern of fattening lambs requires a stable and nutrition-balanced feed supply (Zhong et al., 2018), whereas the traditional loose TMR diet involves substantial labor and animal selection of diet and feed waste (Zhang et al., 2019). In this study, lambs that were fed PTMR had an improved BW, ADG, ADFI, and G/F over the 62-day period compared to those fed UPTMR. Our findings showed an increased TVFA content in the PTMR group when compared to that in the UPTMR group; in particular, the propionate ratio was increased, while butyrate levels and the ratio of acetate to propionate were decreased in the PTMR group. Thus, the feeding of PTMR contributed to greater feed consumption and G/F of lambs and increased the content of VFA compared to that of the UPTMR group. Similarly, Zhong et al. (2018) observed that feeding PTMR with peanut shells as a forage source increased the feed consumption and growth rate of fattening lambs. Li et al. (2021) also revealed that feeding PTMR improved the ADG, ADFI, and ruminal fermentation end products of VFA in lambs. It was documented that the pelleted diet had a higher density than the traditionally loose TMR because of the pelleting process, which resulted in greater feed intake in each bite and less diet occupation in the rumen (Blanco et al., 2014). In addition, VFA, especially propionate, positively influences satiety, endocrine system, glucose homeostasis, and gut barrier integrity and these mechanisms have all been linked to production and animal health. Thus, the augmented total content of ruminal VFA due to PTMR feeding might be another reason to facilitate the increased performance of lambs. In this study, the content of TVFA and propionate proportion also exerted the positive linkage with the increased abundance of microbes in the rumen of lambs fed PTMR diet. This was consistent with the findings of Zhang et al. (2021), who reported that fermentation products in the rumen were related to dietary composition and rumen microbes. The pelleting process denatures proteins, gelatinizes starch, and hydrolyzes some fiber components, all of which contribute to the proliferation of beneficial ruminal microbes (Blanco et al., 2014). In turn, microbes in the rumen determine fermented end products (Petric et al., 2021). This process increases the fermentability of the diet by rumen microorganisms and makes fermentation favorable to produce VFA (Perea et al., 2017). Additionally, previous studies have shown that VFA concentrations are positively correlated with the productivity of animals (Sutton et al., 2003; Calabrò et al., 2005). These support the present findings, with a tight correlation between the increased TVFA, propionate proportions, performance, and ruminal microbes of lambs fed PTMR.

In this study, another interesting finding was that decreased alpha-diversity of ruminal microbiota in the PTMR group than that in the UPTMR group, whereas the differentiated beta-diversity in terms of the separated OPLS-DA distribution between the two groups was observed. Thus, lambs fed with UPTMR had a greater ruminal microbial diversity than those receiving the PTMR diet and the two diets shaped the differential configuration (composition and structure) of ruminal microbiota. In addition, the abundance of *Fibrobacter*, *Rikenellaceae*, and *Veillonellaceae* in rumen was significantly enhanced in the PTMR group compared to that in the UPTMR group. It has been suggested that *Fibrobacter* can produce cellulase and partially degrade the dietary cellulose and hemicellulose to form short-chain fatty acids, thus providing the direct energy source for ruminants (Li et al., 2021). The family *Rikenellaceae* promotes the metabolism of protein and carbohydrate, while *Veillonellaceae* facilitates the intestinal mucosal integrity and augments gut barrier and immune of the intestine (Li et al., 2020; Liu et al., 2021). Moreover, the abundance of *Fibrobacter*, *Rikenellaceae*, and *Veillonellaceae* exhibited a positive correlation to change in performance. In contrast, lambs fed PTMR diet had a decreased abundance of genus *Christensenellaceae*, *Ruminococcaceae*, *Succiniclasticum*, and *Saccharofermentans* compared to those in the UPTMR group and those bacteria negatively correlated with altered performance in terms of BW, ADG, ADFI, and G/F. This result revealed that feeding PTMR diet facilitated the colonization of certain specific beneficial bacteria such as *Fibrobacter*, *Rikenellaceae*, and *Veillonellaceae*, which positively correlated with the improved production performance of fattening sheep.

In this study, OPLS-DA analysis demonstrated two completely separated metabolomes due to the differentiated diet of PTMR and UPTMR and 570 differential metabolites were identified between the two trial groups. Additionally, the Spearman’s rank correlation analysis revealed that variation in increased metabolites (tyrosine, methionine, cysteine, histidine, indole, etc.) in the PTMR group positively correlated with the enhanced performance and abundance of microbes (*Fibrobacter*, *Rikenellaceae*, and *Veillonellaceae*), whereas it was negatively correlated with the increased richness of *Christensenellaceae*, *Ruminococcaceae*, *Succiniclasticum*, and *Saccharofermentans* in the UPTMR group. This indicated that increased microbes in the PTMR group affected the enhanced content of tyrosine, methionine, cysteine, histidine, and indole in the rumen of lambs, which further facilitated the improved performance and G/F of lambs. Similarly, it has been documented that the composition and structure of ruminal microbiota defined the constituents and contents of small molecular metabolites of rumen content (Morgavi et al., 2015; Marie-Etancelin et al., 2021).

The enriched differential metabolic pathways by matching the KEGG database using differentiated metabolites, primarily included amino acids (arginine, tryptophan, histidine, tyrosine, cysteine, and methionine) metabolism and steroid biosynthesis. Among them, the main growth-related pathways involving in metabolisms of tryptophan, histidine, cysteine, methionine, tyrosine, and steroid biosynthesis were upregulated in the PTMR group. Tryptophan, one of the essential amino acids for animals and humans and plays a variety of biological roles in protein biosynthesis, regulating feed intake, alleviating stress, and promoting the growth of animals (Wallace et al., 2019). Thus, increased tryptophan metabolism may be one of the main reasons for the improved production performance. In addition, histidine is a limiting factor for milk protein synthesis and is the first limiting amino acid for the growth of ruminants (Petric et al., 2021). Similarly, cysteine and methionine supplementation facilitate superior performance and protein utilization of calves (Wang et al., 2021). Therefore, the upregulation of amino acid-related metabolic pathways due to the PTMR diet feeding contributes to an increased G/F and production performance of fattening lambs.

Taken together, compared with the UPTMR group, feeding lambs with PTMR characterized the rumen microbiota with a low diversity, but improved richness of certain specific efficient bacteria. Moreover, the abundance of microbes, contents of metabolites, and performance in the PTMR group were strongly correlated. This revealed that the interaction between diet and ruminal microbiota reshaped the exclusive configuration of microbial community, which underlies the alteration of the growth-promoting metabolites and metabolic pathways, which in turn resulted in the elevated production performance of lambs. It has been suggested that an increased enrichment of specific microbes and metabolic pathways, rather than greater diversity, contributed to the better energy harvest and an improved production performance of animals (Bergman, 1990; Shabat et al., 2016). This is consistent with our current results.

The present results support our hypothesis that feeding PTMR altered the ruminal microbiota and metabolome and upregulated the growth-related metabolic pathways, which underlay the increased production performance and G/F. Additionally, this study provides a new viewpoint in understanding the underlying mechanism by which the diet impacts the growth performance of animals through intervening the axis of “microbiota-metabolome-phenotypes” to achieve the superior productivity. The findings also imply that specific growth-related microbes and metabolites may be potential targets for modifying the production performance of animals by specific diet consumption. This provides a novel perspective for developing a dietary strategy to improve the production performance of lambs reared in the intensive feeding system.

In conclusion, compared with those of the UPTMR group, lambs that were fed PTMR had greater production performance and G/F, increased TVFA content, and propionate production in the rumen. Additionally, feeding PTMR characterized the ruminal microbiota with decreased alpha-diversity and increased abundance of specific microbes, including *Fibrobacter*, *Rikenellaceae*, and *Veillonellaceae*, as well as upregulated growth-promoting metabolic pathways primarily involving tryptophan, histidine, cysteine and methionine metabolism, steroid biosynthesis, and tyrosine metabolism. Thus, changes of the ruminal microbiota due to different dietary types underlay the modulation of the metabolome and contribute to an improved production performance, G/F, and rumen fermentation of fattening lambs. Therefore, feeding PTMR to fattening lambs is a feasible strategy for superior production performance in intensive rearing conditions.

## Data Availability Statement

The datasets presented in this study can be found in online repositories. The names of the repository/repositories and accession number(s) can be found below: https://www.ncbi.nlm.nih.gov/, SUB7211899.

## Ethics Statement

The animal study was reviewed and approved by all procedures involving animal care and use were in strict accordance with the animal care and use protocol approved by the Shandong Agricultural University Animal Nutrition Research Institute (Protocol No. 20190316).

## Author Contributions

All authors contributed to intellectual input and assisted with this study and manuscript. GZ and YL designed the experiments and wrote and revised the manuscript. SL and MD conducted the experiments and analyzed the data. CZ and YW performed the determination of samples. All authors read and approved the final version of the manuscript.

## Conflict of Interest

The authors declare that the research was conducted in the absence of any commercial or financial relationships that could be construed as a potential conflict of interest.

## Publisher’s Note

All claims expressed in this article are solely those of the authors and do not necessarily represent those of their affiliated organizations, or those of the publisher, the editors and the reviewers. Any product that may be evaluated in this article, or claim that may be made by its manufacturer, is not guaranteed or endorsed by the publisher.

## Footnotes

1
http://huttenhower.sph.harvard.edu/galaxy/

2
https://www.metaboanalyst.ca/faces/ModuleView.xhtml
