# Supplementary material for: Diet Type Impacts Production Performance of Fattening Lambs by Manipulating the Ruminal Microbiota and Metabolome
Source: Front Microbiol. 2022 Apr 25;13:824001. doi: 10.3389/fmicb.2022.824001 (PMC9081845; doi:10.3389/fmicb.2022.824001)

**Supplementary materials**

**Diet form impacts production performance of fattening lambs by manipulating the** **ruminal microbiome and metabolome**

Siqi Li ^1,^ *, Meiyu Du ^1,^ *, Chongyu Zhang^1^, Yun Wang^1^, Yunkyoung Lee ^2, †^, Guiguo Zhang^1, †^

^1^College of Animal Sciences and Technology, Shandong Agricultural University, 61 Daizong Street, Taian City, Shandong Province, China

^2^ Department of Food Science and Nutrition, Interdisciplinary Graduate Program in Advanced Convergence Technology & Science, Jeju National University, Jeju, Republic of Korea

**† Correspondence:**

Guiguo Zhang [zhanggg@sdau.edu.cn](mailto:zhanggg@sdau.edu.cn)

Yunkyoung Lee [lyk1230@jejunu.ac.kr](mailto:lyk1230@jejunu.ac.kr)

*These authors contributed equally to this study and share the first authorship.

**Table S 1**

Composition and nutrient contents of the experimental diet (DM basis).

| Item | Groups^1^ | |
| --- | --- | --- |
|  | PTMR | UPTMR |
| **Ingredients, g/kg** |  |  |
| Peanut vine | 250 | 250 |
| Leymus chinensis | 150 | 150 |
| Corn | 200 | 200 |
| Soybean meal | 65 | 65 |
| Corn germ meal | 200 | 200 |
| Corn episperm | 90 | 90 |
| Dicalcium phosphate | 3 | 3 |
| Limestone | 3 | 3 |
| Sodium chloride | 4 | 4 |
| Vitamin and trace mineral premix^2^ | 35 | 35 |
| Total | 1000 | 1000 |
| **Nutrient content^3^, g/kg** | | |
| Metabolizable energy (ME), MJ/kg DM^4^ | 11.34 | 11.28 |
| DM | 896 | 878 |
| Crude protein | 147 | 147 |
| Neutral detergent fiber (NDF)^4^ | 326 | 330 |
| Acid detergent fiber (ADF) | 168 | 176 |
| Calcium | 6.68 | 6.71 |
| Total phosphorus (TP) | 4.12 | 3.89 |

^1^PTMR, pelleted total mixed ration; UPTMR, unpelleted total mixed ration.

^2^ Supplied per kilogram of total mixed ration：1367 IU Vitamin A, 194 IU Vitamin D_3_, 15 IU Vitamin E, 74 mg Fe as ferrous sulfate (FeSO_4_ ∙ 7H_2_O), 46.3 mg Zn as zinc sulfate (ZnSO_4_ ∙ 7H_2_O), 36.5 mg Mn as manganese sulfate (MnSO_4_ ∙5H_2_O), 17.0 mg Cu as copper sulfate (CuSO_4_ ∙ 5H_2_O), 1.5 mg I as calcium iodide (KI), 0.3 mg Se as sodium selenite (Na_2_SeO_3_).

^3^ All items of nutrient level except ME were measured values (n = 6), and ME was a calculated value, which was estimated from NRC (2007).

^4^NDF assayed without a heat stable amylase and expressed inclusive of residual ash. ADF expressed inclusive of residual ash.

**Table S 2**

Relative abundance (%) of the ruminal microbiota in phylum, family, and genus levels of lambs receiving PTMR and WPCD diet

| **Items** | **Treatments** | | **SEM** | ***P-values*** |
| --- | --- | --- | --- | --- |
|  | **R.PTMR** | **R.WPCD** |  |  |
| ***Phylum*** |  |  |  |  |
| *Bacteroidetes* | 57.99% | 49.27% | 0.063 | 0.511 |
| *Firmicutes* | 35.13% | 36.06% | 0.041 | 0.912 |
| *Proteobacteria* | 2.51% | 9.12% | 0.025 | 0.226 |
| *Tenericutes* | 0.43% | 2.11% | 0.003 | 0.015 |
| *Synergistetes* | 1.13% | 0.44% | 0.004 | 0.356 |
| *Fibrobacteres* | 1.05% | 0.22% | 0.002 | 0.079 |
| *Actinobacteria* | 0.54% | 0.54% | 0.002 | 0.998 |
| *Cyanobacteria* | 0.03% | 0.34% | 0.001 | 0.295 |
| *Planctomycetes* | 0.14% | 0.34% | 0.001 | 0.307 |
| *Chloroflexi* | 0.01% | 0.22% | 0.001 | 0.134 |
| *Spirochaetes* | 0.39% | 0.32% | 0.001 | 0.592 |
| *Elusimicrobia* | 0.19% | 0.02% | 0.001 | 0.189 |
| **Family** |  |  |  |  |
| *Prevotellaceae* | 41.55% | 29.35% | 0.056 | 0.312 |
| *Veillonellaceae* | 18.39% | 4.16% | 0.043 | 0.138 |
| *Xanthomonadaceae* | 1.77% | 8.09% | 0.024 | 0.222 |
| *Lachnospiraceae* | 10.94% | 8.67% | 0.016 | 0.501 |
| *Muribaculaceae* | 6.83% | 11.83% | 0.023 | 0.307 |
| *Ruminococcaceae* | 3.22% | 11.91% | 0.006 | 0 |
| *Rikenellaceae* | 5.96% | 3.69% | 0.006 | 0.09 |
| *Acidaminococcaceae* | 0.40% | 6.28% | 0.006 | 0.002 |
| *Christensenellaceae* | 0.61% | 2.72% | 0.003 | 0.004 |
| *Synergistaceae* | 1.13% | 0.44% | 0.004 | 0.356 |
| *Fibrobacteraceae* | 1.05% | 0.22% | 0.002 | 0.079 |
| *unidentified_Clostridiales* | 0.68% | 1.10% | 0.002 | 0.267 |
| *unidentified_Cyanobacteria* | 0.03% | 0.34% | 0.001 | 0.295 |
| *Atopobiaceae* | 0.38% | 0.01% | 0.001 | 0.216 |
| *unidentified_Bacteroidales* | 0.46% | 0.50% | 0.001 | 0.858 |
| *Erysipelotrichaceae* | 0.57% | 0.51% | 0.001 | 0.821 |
| *Desulfobulbaceae* | 0.30% | 0.07% | 0.001 | 0.326 |
| *Nocardiaceae* | 0.04% | 0.25% | 0.001 | 0.329 |
| *Succinivibrionaceae* | 0.03% | 0.33% | 0.001 | 0.104 |
| *Anaerolineaceae* | 0.01% | 0.20% | 0.001 | 0.146 |
| *Spirochaetaceae* | 0.39% | 0.32% | 0.001 | 0.592 |
| *Endomicrobiaceae* | 0.19% | 0.02% | 0.001 | 0.189 |
| **Genus** |  |  |  |  |
| *Quinella* | 13.95% | 2.81% | 0.048 | 0.277 |
| *unidentified_Prevotellaceae* | 12.89% | 8.19% | 0.022 | 0.316 |
| *Stenotrophomonas* | 1.77% | 8.08% | 0.024 | 0.222 |
| *Succiniclasticum* | 0.37% | 6.24% | 0.006 | 0.002 |
| *unidentified_Ruminococcaceae* | 1.06% | 6.56% | 0.003 | 0 |
| *Fretibacterium* | 1.13% | 0.43% | 0.004 | 0.356 |
| *unidentified_Lachnospiraceae* | 1.65% | 1.82% | 0.003 | 0.768 |
| *unidentified_Veillonellaceae* | 0.89% | 0.19% | 0.003 | 0.2 |
| *Saccharofermentans* | 0.23% | 1.23% | 0.002 | 0.029 |
| *Fibrobacter* | 1.05% | 0.22% | 0.002 | 0.079 |
| *unidentified_Cyanobacteria* | 0.03% | 0.34% | 0.001 | 0.295 |
| *Olsenella* | 0.34% | 0.00% | 0.001 | 0.241 |
| *unidentified_Bacteroidales* | 0.46% | 0.50% | 0.001 | 0.858 |
| *Anaerovibrio* | 0.38% | 0.24% | 0.001 | 0.558 |
| *Desulfobulbus* | 0.30% | 0.07% | 0.001 | 0.326 |
| *unidentified_Rikenellaceae* | 0.66% | 0.33% | 0.001 | 0.064 |
| *Rhodococcus* | 0.04% | 0.25% | 0.001 | 0.329 |
| *unidentified_Clostridiales* | 0.15% | 0.35% | 0.001 | 0.291 |
| *Acetitomaculum* | 0.21% | 0.63% | 0.001 | 0.053 |
| *Kandleria* | 0.27% | 0.00% | 0.001 | 0.183 |
| *Moryella* | 0.07% | 0.31% | 0.001 | 0.135 |
| *Syntrophococcus* | 0.20% | 0.04% | 0.001 | 0.319 |
| *Flexilinea* | 0.01% | 0.20% | 0.001 | 0.145 |
| *unidentified_Christensenellaceae* | 0.01% | 0.26% | 0.001 | 0.053 |
| *Anaerovorax* | 0.20% | 0.24% | 0.001 | 0.739 |
| *Pseudobutyrivibrio* | 0.04% | 0.18% | 0.001 | 0.247 |
| *Candidatus_Endomicrobium* | 0.19% | 0.02% | 0.001 | 0.189 |
| *Oribacterium* | 0.28% | 0.17% | 0.000 | 0.241 |

**Table S2** Different metabolites between PTMR and UPTMR groups with LC-MS/MS (ESI+)

| **Name_des** | **log2FC** | ***P*-value** | **VIP** | **Up.Down** |
| --- | --- | --- | --- | --- |
| Coniferyl ferulate | 8.439309 | 1.26E-11 | 5.019847 | up |
| (-)-8-Prenylnaringenin | 8.052045 | 1.65E-09 | 4.778326 | up |
| 8-Iso-15-keto-prostaglandin-F2β | 3.25617 | 7.68E-09 | 1.937471 | up |
| Brazilein | 4.538976 | 2.28E-08 | 2.707454 | up |
| Nigakilactone N | -4.49851 | 3.61E-08 | 2.675427 | down |
| Homaline | 5.607164 | 4.12E-08 | 3.32241 | up |
| Seneciphylline | 3.702676 | 5.83E-08 | 2.193857 | up |
| Phthalin | 3.387714 | 9.08E-08 | 2.018326 | up |
| 2(3H)-Furanone, dihydro-3,4-divanillyl- | 6.72596 | 1.12E-07 | 3.969551 | up |
| Gabapentin enacarbil | 3.993723 | 1.72E-07 | 2.368117 | up |
| Wikstromol | 5.093142 | 2.42E-07 | 3.02019 | up |
| Tangeritin | 4.734747 | 3.78E-07 | 2.790562 | up |
| Alangimarine | 3.211536 | 3.97E-07 | 1.899854 | up |
| Ocaperidone | 3.076372 | 4.00E-07 | 1.824923 | up |
| 17beta-Hydroxy-7alpha-mercaptoandrost-4-en-3-one 7-propionate | 3.174797 | 4.12E-07 | 1.877397 | up |
| p-Toluenesulfonic acid | -2.34741 | 5.53E-07 | 1.399961 | down |
| Ubiquinone Q4 | 3.582226 | 1.17E-06 | 2.118709 | up |
| Eugenitin | 2.414519 | 1.39E-06 | 1.442915 | up |
| N-Phenylacetyl pyroglutamic acid | 3.510105 | 1.46E-06 | 2.081976 | up |
| Perindoprilat | 4.011779 | 2.35E-06 | 2.358649 | up |
| Brassylic acid | -4.52319 | 2.39E-06 | 2.720379 | down |
| Ethinylestradiol sulfonate | 2.335831 | 2.40E-06 | 1.396056 | up |
| Ilepcimide | 3.612077 | 3.21E-06 | 2.168739 | up |
| Troxipide | 3.790273 | 3.57E-06 | 2.222828 | up |
| Octamethyltrisiloxane | 2.140761 | 3.58E-06 | 1.267071 | up |
| Byakangelicol | 4.528239 | 3.68E-06 | 2.743804 | up |
| Saccharin | 5.634675 | 3.71E-06 | 3.298086 | up |
| MFCD00075988 | 5.57084 | 4.29E-06 | 3.35989 | up |
| 4,4-Bis[4-(acetyloxy)phenyl]3-hexanone | 3.468724 | 4.34E-06 | 2.042993 | up |
| Xanthohumol | 4.907345 | 5.22E-06 | 2.883132 | up |
| Methyl nigakinone | -3.84264 | 6.12E-06 | 2.27291 | down |
| yatein | 2.407024 | 6.57E-06 | 1.417882 | up |
| Isopimpinellin | -3.56906 | 7.28E-06 | 2.144006 | down |
| Maclurin | -3.76278 | 7.52E-06 | 2.207201 | down |
| CB5740000 | 3.08994 | 9.88E-06 | 1.816139 | up |
| Leukotriene E4 | -3.48321 | 1.03E-05 | 2.05289 | down |
| Roxatidine | 3.199304 | 1.13E-05 | 1.87814 | up |
| prosolanapyrone II | 4.294855 | 1.15E-05 | 2.521796 | up |
| Khellin | 3.159344 | 1.41E-05 | 1.910519 | up |
| Heliettin | 5.593689 | 1.43E-05 | 3.276069 | up |
| Osthol | -3.60561 | 1.49E-05 | 2.195266 | down |
| 2-Ethylhexyl hydrogen sulfate | 3.212555 | 1.57E-05 | 1.942639 | up |
| Testosterone glucuronide | -2.6317 | 1.58E-05 | 1.574304 | down |
| Entinostat | 2.043258 | 1.64E-05 | 1.22879 | up |
| 2,2-Dimethyl-8-prenylchromene 6-carboxylic acid | -2.99088 | 1.74E-05 | 1.7532 | down |
| Cinobufagin | 3.497389 | 1.75E-05 | 2.047208 | up |
| S-Adenosyl-L-methionine | 4.073084 | 1.88E-05 | 2.374585 | up |
| ANGOLENSIN (R) | 7.355575 | 1.94E-05 | 4.245919 | up |
| cinnamodial | 3.535671 | 1.95E-05 | 2.065841 | up |
| Rutacridone epoxide | -1.939 | 2.00E-05 | 1.148624 | down |
| Punctaporonin B | -2.13843 | 2.19E-05 | 1.278258 | down |
| Enoxacin | 2.638857 | 3.08E-05 | 1.570998 | up |
| Chloramphenicol stearate | -2.0525 | 3.12E-05 | 1.207432 | down |
| Monobenzone | -2.94069 | 3.24E-05 | 1.715697 | down |
| Acetylshikonin | 4.374679 | 3.60E-05 | 2.68007 | up |
| 3-Hydroxybenzoic acid | 3.645011 | 3.73E-05 | 2.183045 | up |
| amfonelic acid | -2.56674 | 3.74E-05 | 1.506448 | down |
| Methyl Red | -2.14908 | 3.86E-05 | 1.287726 | down |
| Lunacalcipol | -2.32817 | 3.94E-05 | 1.376609 | down |
| chloromebuform | 2.176843 | 4.42E-05 | 1.275726 | up |
| licochalcone B | 5.783241 | 4.67E-05 | 3.315073 | up |
| 1-(2,6-Dihydroxy-4-methoxy-3,5-dimethylphenyl)-3-phenyl-1-propanone | -3.3085 | 4.80E-05 | 1.954458 | down |
| Portulacaxanthin I | 4.673631 | 4.87E-05 | 2.718769 | up |
| indole-3-propanol phosphate | 4.497864 | 4.96E-05 | 2.624274 | up |
| 6'-Methoxycinchonan-3,9-diol | 2.186598 | 5.24E-05 | 1.278429 | up |
| protoporphyrinogen | -2.71791 | 5.28E-05 | 1.58831 | down |
| 3-Acetylnerbowdine | -3.11952 | 5.53E-05 | 1.896638 | down |
| Glaucarubin | -3.70985 | 5.54E-05 | 2.257734 | down |
| etifenin | 2.323881 | 5.87E-05 | 1.363987 | up |
| melicopicine | 2.249984 | 6.37E-05 | 1.330547 | up |
| 5-(6-Hydroxy-benzofuran-2-yl)-2-(3-methyl-but-2-enyl)-benzene-1,3-diol | 2.474473 | 6.84E-05 | 1.46482 | up |
| 2-Amino-9,10-epoxy-8-oxodecanoic acid | -4.62933 | 7.88E-05 | 2.882955 | down |
| Phaseolin | 5.80326 | 8.06E-05 | 3.345128 | up |
| 2-[2-(3,4-dimethoxyphenyl) ethyl]-4-methoxy-2,3-dihydropyran-6-one | -2.12203 | 8.37E-05 | 1.256753 | down |
| gibberellin A7 | 2.552806 | 8.55E-05 | 1.558263 | up |
| Croconazole | 2.2438 | 8.68E-05 | 1.367445 | up |
| DO0750000 | -2.41508 | 8.82E-05 | 1.46294 | down |
| BIM-1 | -2.38644 | 9.12E-05 | 1.442685 | down |
| 2'-Methoxyformonetin | 7.157314 | 0.0001 | 3.970912 | up |
| Budesonide | 2.114627 | 0.000112 | 1.245247 | up |
| callystatin A | 5.099325 | 0.000117 | 2.928358 | up |
| Quillaic Acid | -2.07043 | 0.000121 | 1.244762 | down |
| Compactin | -2.21517 | 0.000124 | 1.326452 | down |
| icomucret | -2.67281 | 0.000126 | 1.585638 | down |
| Hydroxycarteolol | 3.453248 | 0.000126 | 2.00355 | up |
| Kaempferol | 3.808678 | 0.000139 | 2.190425 | up |
| IpA | -1.74249 | 0.000166 | 1.054049 | down |
| Prostaglandin F2α 1-11-lactone | -2.16763 | 0.000201 | 1.324057 | down |
| 2-(2-Acetoxy-2-oxoethyl)-2-hydroxysuccinate | -3.25315 | 0.000204 | 2.004462 | down |
| Tricetamide | 2.061144 | 0.000214 | 1.194147 | up |
| atrovirinone | 2.716228 | 0.000214 | 1.566476 | up |
| Acronycidine | 2.318905 | 0.000236 | 1.350292 | up |
| Sofalcone | -2.52788 | 0.000245 | 1.539332 | down |
| (E)-3,4,5-Trimethoxycinnamic acid | 2.40501 | 0.000245 | 1.387066 | up |
| Benzonatate | 2.388928 | 0.000266 | 1.472772 | up |
| ascorbic acid 2-sulfate | 3.360906 | 0.000279 | 1.923255 | up |
| Salvianolic acid A | -2.30668 | 0.000281 | 1.395841 | down |
| chelirubine | 2.174623 | 0.000286 | 1.25738 | up |
| 3-sulfolactic acid | 2.414775 | 0.000303 | 1.437329 | up |
| Oxypeucedanin | 3.985712 | 0.000304 | 2.256271 | up |
| Dibenzo-18-crown-6 | 2.52433 | 0.000306 | 1.573437 | up |
| Glycyrin | 2.878039 | 0.000312 | 1.647684 | up |
| 3-O-(alpha-L-olivosyl)oleandolide | -2.7001 | 0.000324 | 1.564513 | down |
| 20-Hydroxy-(5Z,8Z,11Z,14Z)-eicosatetraenoic acid | -2.74368 | 0.000327 | 1.656214 | down |
| Ptaquiloside | 2.211297 | 0.000339 | 1.314028 | up |
| Mubritinib | -4.86071 | 0.00038 | 2.689142 | down |
| 1,7-bis(4-hydroxyphenyl)heptan-3-one | 6.226456 | 0.000394 | 3.441341 | up |
| Zinnolide | -2.3549 | 0.000395 | 1.458416 | down |
| Sesamex | -2.43313 | 0.0004 | 1.397942 | down |
| Homovanillic acid | 2.615895 | 0.000403 | 1.501783 | up |
| Linetastine | 2.885388 | 0.000419 | 1.646149 | up |
| Garcinol | 2.16781 | 0.00044 | 1.259298 | up |
| Retosiban | -3.13867 | 0.000442 | 1.982125 | down |
| 3,22-Dihydroxy-28-oxoolean-12-en-16-yl acetate | 1.977432 | 0.000474 | 1.144924 | up |
| Schisandrin C | 1.846648 | 0.000477 | 1.065462 | up |
| Casimiroin | -1.82383 | 0.000478 | 1.1151 | down |
| Hexyl 2-furoate | 1.637192 | 0.000488 | 1.000954 | up |
| Trenbolone Acetate | 5.06726 | 0.000506 | 2.846436 | up |
| MFCD00083068 | -3.86668 | 0.000525 | 2.576195 | down |
| ONONETIN | -1.97189 | 0.000532 | 1.203808 | down |
| (E)-4-Methoxycinnamic acid | -2.47721 | 0.00061 | 1.520768 | down |
| Knipholone | -2.60387 | 0.000613 | 1.477703 | down |
| Resveratrol | -2.5267 | 0.000653 | 1.457236 | down |
| 6 beta hydroxy testosterone | -1.82711 | 0.000683 | 1.112269 | down |
| Phaseic acid | 2.219071 | 0.000722 | 1.274377 | up |
| Taxifolin | 2.996065 | 0.000739 | 1.687348 | up |
| (+/-)-hesperetin | 3.604432 | 0.00074 | 2.03769 | up |
| Paliperidone | -2.03708 | 0.000745 | 1.161933 | down |
| (-)-licarin A | 5.683978 | 0.000766 | 3.08071 | up |
| Nonanoic acid | -2.02348 | 0.000784 | 1.252947 | down |
| Sacubitril | -1.88489 | 0.000785 | 1.176163 | down |
| (-)-Andrographolide | -3.10846 | 0.000793 | 2.021623 | down |
| N-tert-Butyloxycarbonyl-deacetyl-leupeptin | 1.801303 | 0.000805 | 1.040363 | up |
| Tilnoprofen arbamel | 1.754536 | 0.000831 | 1.02724 | up |
| Niaprazine | -2.4286 | 0.000864 | 1.546665 | down |
| Pruvanserin | 1.840087 | 0.000886 | 1.052452 | up |
| nummularine F | -3.27428 | 0.000954 | 2.089499 | down |
| MFCD00143247 | -3.17939 | 0.000999 | 1.739991 | down |
| Metopimazine | 2.14973 | 0.001148 | 1.211779 | up |
| 2-Isocapryloyl-3R-hydroxymethyl-gamma-butyrolactone | -2.6866 | 0.001163 | 1.728059 | down |
| Deferoxamine | 2.401008 | 0.00119 | 1.521918 | up |
| Saikosaponin BK1 | 5.02181 | 0.001197 | 2.640378 | up |
| NOP | -2.23416 | 0.001216 | 1.252993 | down |
| (-)-Chorismic acid | -2.87929 | 0.001238 | 1.647536 | down |
| 4-methoxy-9,10-dihydrophenanthrene-2,7-diol | -1.77978 | 0.001244 | 1.06994 | down |
| Candesartan | 2.308166 | 0.001247 | 1.296776 | up |
| Oxazolam | 2.495243 | 0.00126 | 1.592992 | up |
| Diosmetin | 5.895448 | 0.001418 | 3.013361 | up |
| tricoumaroyl spermidine | 2.612273 | 0.001462 | 1.449755 | up |
| Methdilazine | 3.195139 | 0.001479 | 1.772597 | up |
| epi-Tulipinolide | -2.39195 | 0.001485 | 1.447817 | down |
| obacunone | 1.762084 | 0.001547 | 1.007274 | up |
| Fisetin | 4.379454 | 0.00155 | 2.332314 | up |
| Eldecalcitol | -2.03184 | 0.001682 | 1.287436 | down |
| beta-D-Fructofuranosyl 4-O-(2-methylbutanoyl)-alpha-D-glucopyranoside | 1.632772 | 0.001775 | 1.013734 | up |
| 15S-hydroxyeicosatrienoic acid | -2.18173 | 0.001864 | 1.397718 | down |
| MFCD00055031 | 2.012209 | 0.001952 | 1.209333 | up |
| Melengestrol acetate | -2.94409 | 0.002077 | 2.031214 | down |
| Asiaticoside | 5.463502 | 0.002082 | 2.901507 | up |
| Telapristone acetate | -2.65892 | 0.002175 | 1.766838 | down |
| 1-hexadecanoyl-2-octadecanoyl-sn-glycero-3-phospho-(1'-sn-glycerol) | 2.886192 | 0.002286 | 1.599247 | up |
| Cuauhtemone | -2.61379 | 0.002299 | 1.761953 | down |
| Mitraphylline | 4.167379 | 0.002389 | 2.278951 | up |
| DL-Mandelic acid | 2.861566 | 0.002402 | 1.5773 | up |
| fenalamide | -1.93537 | 0.00255 | 1.224804 | down |
| Equol | -1.599 | 0.002742 | 1.021541 | down |
| Crocetin | 3.935684 | 0.003084 | 2.079 | up |
| Loxoprofen | -2.46848 | 0.003293 | 1.69758 | down |
| EUGENYL GLUCOSIDE | -2.67414 | 0.003342 | 1.651501 | down |
| MFCD02178588 | 3.300217 | 0.003445 | 1.784086 | up |
| 2-Oxo-3-(3-pyridinyl)-2H-chromen-7-yl acetate | -2.11896 | 0.003554 | 1.442809 | down |
| N-Phenyl-beta-D-glucopyranosylamine | 3.311769 | 0.003965 | 1.790603 | up |
| Mucronine B | -2.35584 | 0.00416 | 1.609178 | down |
| 13-KODE | -3.30656 | 0.004269 | 1.964088 | down |
| Brivanib | -1.78471 | 0.004337 | 1.190436 | down |
| n-Butyl lactate | 5.58775 | 0.004344 | 2.845985 | up |
| Casticin | -2.88802 | 0.004397 | 1.85105 | down |
| SYSU-00437 | 5.897424 | 0.00442 | 2.791159 | up |
| Calcimycin | -3.4331 | 0.004491 | 2.469369 | down |
| Cystothiazole A | 2.021859 | 0.004533 | 1.105637 | up |
| cromoglicic acid | 4.700747 | 0.004702 | 2.47212 | up |
| Exserohilone | -1.78606 | 0.00475 | 1.089964 | down |
| Omadacycline | 2.376827 | 0.004795 | 1.315475 | up |
| marrubin | -2.46428 | 0.004967 | 1.673178 | down |
| Hydrocortisone Valerate | -2.289 | 0.005107 | 1.57134 | down |
| stylisterol A | -2.87156 | 0.005415 | 1.977264 | down |
| 6,8-Dihydroxy-3-[(2S)-2-hydroxy-4-oxopentyl]-1H-isochromen-1-one | 2.583373 | 0.005448 | 1.409726 | up |
| Tetradecanedioic acid | 2.487847 | 0.005553 | 1.626923 | up |
| 2,6-Bis(3,4-methylenedioxyphenyl)-3,7-dioxabicyclo(3.3.0)octane | 4.469368 | 0.005668 | 2.145495 | up |
| osajin | 3.778432 | 0.005874 | 1.90886 | up |
| Medicarpin 3-O-glucoside-6'-malonate | 4.369255 | 0.006004 | 2.319094 | up |
| DEHYDROCOSTUS LACTONE | -2.93095 | 0.006033 | 2.490573 | down |
| Vorinostat | -2.11098 | 0.006878 | 1.167892 | down |
| N~6~-Octanoyllysine | 2.325391 | 0.007326 | 1.258118 | up |
| Ginsenoside Ro | 2.782599 | 0.007803 | 1.498635 | up |
| Ninhydrin | 3.075897 | 0.007814 | 1.645598 | up |
| (-)-Acutumine | 3.392733 | 0.007912 | 1.784184 | up |
| Zuclopenthixol decanoate | -2.3149 | 0.007974 | 1.290489 | down |
| Parietin | 7.315191 | 0.008117 | 3.355924 | up |
| docarpamine | -1.79423 | 0.008157 | 1.250081 | down |
| oxandrolone | -2.09525 | 0.009401 | 1.502494 | down |
| Escin IB | -2.9032 | 0.009553 | 1.462497 | down |
| 13,14-Dihydro-15-keto Prostaglandin J2 | -3.2665 | 0.010017 | 2.260765 | down |
| Stiripentol | -1.95106 | 0.010036 | 1.462014 | down |
| gaboxadol | 2.617781 | 0.010044 | 1.379658 | up |
| Lupulone | 2.079819 | 0.010387 | 1.145288 | up |
| Rottlerin | -4.2696 | 0.010721 | 1.973452 | down |
| zoxazolamine | 2.507592 | 0.010998 | 1.262646 | up |
| Chryso-obtusin glucoside | 3.113205 | 0.011279 | 1.540637 | up |
| Anhweidelphinine | 4.30165 | 0.011413 | 2.113734 | up |
| 2′-Hydroxy-4,4′,6′-trimethoxychalcone | -1.79363 | 0.011936 | 1.327349 | down |
| Patidegib | -1.97347 | 0.012075 | 1.616244 | down |
| Sulindac | -2.07357 | 0.012334 | 1.547906 | down |
| CD-1790 | 4.161748 | 0.012507 | 2.071602 | up |
| tarazepide | 1.736298 | 0.013661 | 1.244262 | up |
| Mazaticol | 5.717137 | 0.013757 | 2.418033 | up |
| UNII:Z5JO63XGNK | -1.76954 | 0.01457 | 1.437011 | down |
| Phenanthrene-4,5-dicarboxylate | 4.187819 | 0.014809 | 2.143992 | up |
| Furomine | -1.99577 | 0.015372 | 1.034305 | down |
| Patuletin | 2.459861 | 0.017094 | 1.237402 | up |
| GW9662 | 2.820845 | 0.017559 | 1.598457 | up |
| Aklomide | 2.540157 | 0.018195 | 1.254327 | up |
| 6-(1,2,3,4-Tetrahydro-6-methoxy-2-naphthyl)-2(1H)-pyridone | 2.223212 | 0.021055 | 1.178293 | up |
| 1-oleoyl-2-arachidonoyl-sn-glycero-3-phospho-L-serine | 2.138136 | 0.022081 | 1.094547 | up |
| Val-Trp | -1.81692 | 0.022367 | 1.47279 | down |
| 6-O-[Bis(diisopropylamino)acetyl]hexonic acid | 2.789628 | 0.02448 | 1.41539 | up |
| Nilestriol | -1.95709 | 0.024499 | 1.604981 | down |
| flumedroxone | -2.6259 | 0.024537 | 1.437728 | down |
| 16-Heptadecyne-1,2,4-triol | 2.043743 | 0.025305 | 1.040512 | up |
| 3,16,21,28-Tetrahydroxyolean-12-en-22-yl (2Z)-2-methyl-2-butenoate | 2.469541 | 0.02576 | 1.156794 | up |
| hercynylcysteine sulfoxide | 2.928698 | 0.026587 | 1.550605 | up |
| S-(4-Azidophenacyl)glutathione | 2.041519 | 0.026688 | 1.01059 | up |
| Euxanthone | 5.218861 | 0.027645 | 2.147108 | up |
| N-Heptadecanoylglycine | 3.578695 | 0.02867 | 1.722086 | up |
| Mocetinostat | 2.494094 | 0.031444 | 1.129024 | up |
| Caprylic acid | -1.21018 | 0.031678 | 1.007618 | down |
| bremelanotide | 1.961778 | 0.031714 | 1.080732 | up |
| Genistein | -2.20648 | 0.032662 | 1.205362 | down |
| Lauric acid | -1.3316 | 0.033085 | 1.363269 | down |
| Rosmarinine | 2.299622 | 0.033305 | 1.091598 | up |
| geranyl-PP | -1.92783 | 0.03332 | 1.104885 | down |
| hesperetin 7-O-beta-D-glucoside | 2.669966 | 0.036632 | 1.233199 | up |
| Soyasaponin I | 2.318264 | 0.036818 | 1.766598 | up |
| 5-(4-acetoxybut-1-ynyl)-2,2'-bithiophene | 2.010648 | 0.0388 | 1.062983 | up |
| Vomicine | 4.038258 | 0.03996 | 1.695388 | up |
| Sophoramine | 2.99852 | 0.041478 | 1.580316 | up |
| Proclonol | -3.86238 | 0.044034 | 1.674731 | down |
| Silandrone | 2.454206 | 0.045036 | 1.340722 | up |
| ON1225000 | -1.71288 | 0.046892 | 1.030267 | down |
| gypsogenin 3-O-rhamnosylglucosiduronic acid | 2.193289 | 0.047912 | 1.010665 | up |
| (+)-Alantolactone | -1.30946 | 0.047932 | 1.423691 | down |
| Aurasperone A | 3.118009 | 0.048162 | 1.255452 | up |
| SB-206553 | 2.069824 | 0.049374 | 1.129049 | up |

**FIGURE 1∣** Experimental design of fattening lambs fed with different TMR diets.


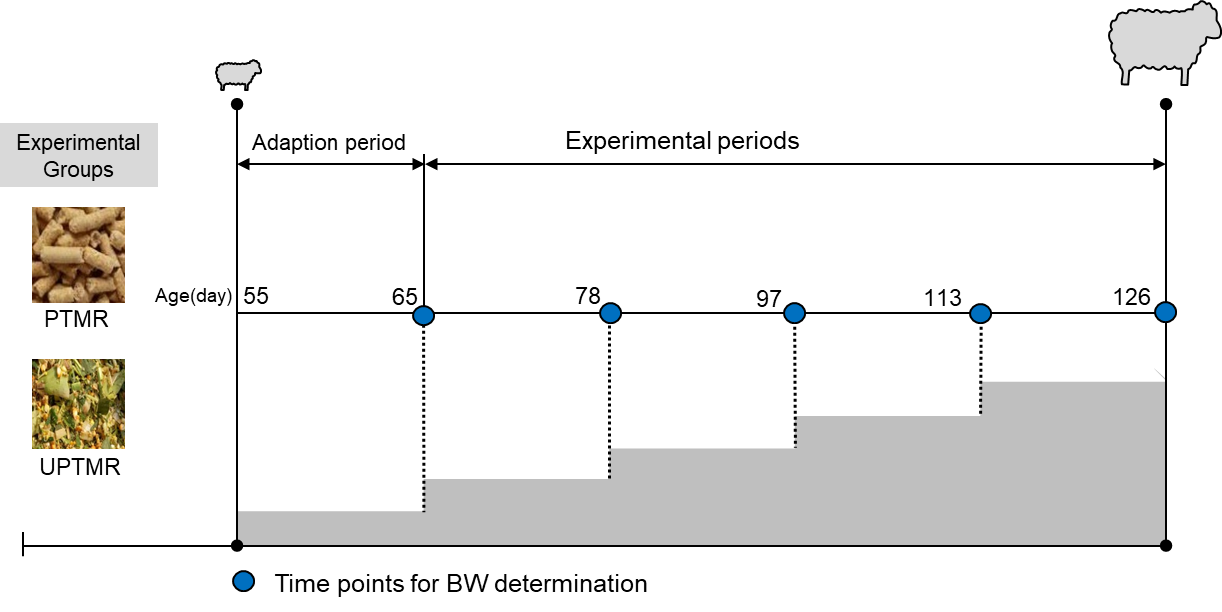

Supplement: Supplementary file 1 [file Data_Sheet_1.docx]
